# New insights into the regulatory role of microRNA in tumor angiogenesis and clinical implications

**DOI:** 10.1186/s12943-018-0766-4

**Published:** 2018-02-07

**Authors:** Ye Wang, Liya Wang, Cheng Chen, Xiaoyuan Chu

**Affiliations:** 1 Department of Medical Oncology, Jinling Hospital, Nanjing Clinical School of Southern Medical University, 305 East Zhongshan Road, Nanjing, 210002 Jiangsu China; 2 Department of Medical Oncology, Jinling Hospital, School of Medicine, Nanjing University, 305 East Zhongshan Road, Nanjing, Jiangsu 210002 China

**Keywords:** Tumor angiogenesis, microRNA, Extracellular vesicle, Biomarker, miRNA delivery

## Abstract

Angiogenesis is essential for tumor growth and metastasis. Understanding the regulation of tumor angiogenesis has become increasingly important. MicroRNAs (miRNAs) are small noncoding RNAs that function in diverse biological processes via post-transcriptional regulation. Extensive studies have revealed two important regulatory roles of miRNAs in tumor angiogenesis: miRNAs in tumor cells affect the activity of endothelial cells via non-cell-autonomous mechanisms, and miRNAs in endothelial cells regulate the cell-autonomous behavior. Recent advances have further highlighted the role of tumor-derived extracellular vesicles in the regulation of tumor angiogenesis via transferring miRNAs to endothelial cells. In this review, we summarize the regulatory role of miRNA in tumor angiogenesis, with a highlight on clinical implications of miRNAs as biomarkers for anti-angiogenic therapy response, and as therapeutic interventions against tumor angiogenesis in vivo.

## Background

Inducing angiogenesis is one of the hallmarks of cancer [1]. Tumor-associated neovasculature is important for delivering nutrients and oxygen to growing tumors, and also playing key roles in multi-aspect of tumor biology, including tumor dissemination/metastasis [2], metabolic deregulation [3] and cancer stem cell maintenance [4, 5]. For its fundamental role in tumor growth and progression, angiogenesis has become an appealing target in cancer treatment.

Angiogenesis is a complex process by which new blood vessels are formed from pre-existing ones by sprouting, remodeling and expansion of primary vascular networks [6]. In normal conditions, following this morphogenesis, the vasculature becomes largely quiescent. However, within tumors, an “angiogenic switch” is always activated, causing continuous generation of new vessels [7]. The “angiogenic switch” is governed by pro-angiogenic and anti-angiogenic signals elicited by tumor cells or tumor microenvironment [8, 9]. When pro-angiogenic signals are activated or anti-angiogenic signals are inhibited, the “angiogenic switch” is turned on. Understanding the regulation of tumor angiogenesis is critical to developing therapeutic strategies against cancer.

MicroRNAs (miRNAs) are small non-coding RNAs [10], that negatively regulate gene expression at post-transcriptional level. Through sequence-specific interaction with the 3′-untranslated region (UTR) of target mRNA, miRNAs suppress gene expression via transcript degradation or inhibition of protein translation. To date, miRNAs have been reported to play key roles in diverse biological processes of cancer, including tumor growth, metastasis, angiogenesis and drug resistance [11–14]. Extensive studies have shown that miRNAs could modulate the function of endothelial cells (ECs) via non-cell-autonomous (genetic and epigenetic alterations in a cell affect the phenotypes of other cells through changing microenvironment or intercellular interaction) and cell-autonomous (genetic and epigenetic alterations in a cell affect the cell’s own phenotypes) manner during tumor angiogenesis. MiRNAs in tumor cells regulate the expression of pro- or anti-angiogenic factors, thereby modulating the proliferation and migration of ECs in a paracrine manner. Endothelial miRNAs regulate the response of ECs to multiple angiogenic stimuli mainly by targeting growth factor receptors and signaling molecules autonomously. Recently, extracellular vesicles (EVs) are emerging as mediators of intercellular communication [15]. Cancer cell-derived miRNA-containing EVs could be transferred to ECs where they induce pro-angiogenic effects. Importantly, several miRNAs have been shown to serve as predictive biomarkers for anti-angiogenic therapy response, especially as non-invasive biomarkers in the circulation. Furthermore, with the development of RNA delivery technology, miRNA-based interventions may act as novel therapeutic means to target tumor angiogenesis.

In our review, we will summarize the non-cell-autonomous and cell-autonomous roles of miRNAs, as well as EV-transferred miRNAs in the regulation of tumor angiogenesis. Moreover, we highlight the clinical implications of miRNAs as predictive biomarkers for anti-angiogenic therapy response in both tissue and circulation, and several miRNA delivery approaches that have been applied to suppress angiogenesis in vivo.

## Non-cell-autonomous regulation of tumor angiogenesis by miRNAs in tumor cells

Angiogenesis is under the control of pro-angiogenic and anti-angiogenic factors [7]. Tumor cells could produce and secrete such factors into surrounding environment to promote vessel growth. Endogenous activators and inhibitors are frequently targeted in tumor cells by miRNAs, which thereby regulate angiogenesis in a non-cell-autonomous manner (Table 1).

**Table 1:** MiRNAs involved in non-cell-autonomous regulation of tumor angiogenesis in tumor cells

Targets	miRNAs	Expression in tumor cells	Ref
VEGF	miR-20, miR-29b, miR-93, miR-126, miR-190, miR-195, miR-200, miR-203, miR-497, miR-503, miR-638, miR-27b, miR-128	down	[21–42]
VEGF inducers			
HIF-1	miR-22, miR-107, miR-519c	down	[43–45]
HIF-2	miR-145	down	[46]
PI3K/AKT pathway	miR-26a, miR-145	down	[47, 48]
mTOR pathway	miR-18a, miR-128, miR-145, miR-218	down	[49–52]
IGF1R pathway	miR-126, miR-181b, miR-148a, miR-152	down	[53–55]
VEGF upstream TFs			
STAT3	miR-874	down	[56]
Bmi-1	miR-16	down	[57]
E2F3	miR-34a	down	[58]
NF90	miR-590-5p	down	[59]
VEGF suppressor			
PTEN	miR-21	up	[60]
E-cadherin	miR-9	up	[61]
VEGF independent factors			
FGF	miR-503	down	[38]
HDGF	miR-214, miR-497	down	[62, 63]
angiogenin	miR-409-3p	down	[64]
angiopoietin	miR-204	down	[65]
MMPs	miR-9, miR-26b-5p, miR-98, miR-181a-5p	Down	[66–69]
TSP-1	miR-17-92 cluster, miR-182, miR-194, miR-467	up	[75–78]

*IGF1R* Insulin-like growth factor I receptor, *Bmi-1* B-cell-specific Moloney murine leukemia virus integration site 1, *E2F3* E2F transcription factor 3, *NF90* Nuclear factor 90, *TSP-1* thrombospondin-1, *HDGF* hepatoma-derived growth factor

### Tumoral miRNAs regulating VEGF-dependent angiogenesis

The most predominant growth factor of angiogenesis is VEGF, consisting of VEGF-A/B/C/D and placental growth factor (PlGF) [16, 17]. VEGF-A (also known as VEGF) is a potent trigger for angiogenesis by signaling through VEGF receptor-2 (VEGFR-2) [18]. While VEGF-B specifically regulate the embryonic angiogenesis of myocardial tissue [19] and VEGF-C/D mainly participates in the genesis and maintenance of lymphatic vessels. Therefore, among the VEGF factors, VEGF-A is most widely studied in the regulation of tumor angiogenesis [20].

MiR-20 [21], miR-29b [22], miR-93 [23–25], miR-126 [26–30], miR-190 [31], miR-195 [32], miR-200 [33, 34], miR-203 [35], miR-497 [36, 37], miR-503 [38] and miR-638 [39] have been experimentally shown to directly target the 3’UTR region of VEGF-A mRNA in different types of cancer and subsequently impairing the pro-angiogenesis signaling of VEGF/VEGFR-2 in endothelial cells. These miRNAs are generally downregulated in cancer. Recently, the role of VEGF-C in initiating and potentiating neo-angiogenesis has been uncovered [40]. MiR-27b and miR-128 functioned as inhibitor of tumor progression and angiogenesis through targeting VEGF-C [41, 42].

Apart from directly targeting VEGF, a handful of miRNAs regulate VEGF-dependent tumor angiogenesis by targeting VEGF inducers, such as HIF pathway (miR-22 [43], miR-107 [44], miR-519c [45], miR-145 [46]), PI3K/AKT pathway (miR-26a [47], miR-145 [48]), mTOR pathway (miR-18a [49], miR-128 [50], miR-145 [51], miR-218 [52]), IGF1R pathway (miR-126 [53], miR-181b [54], miR-148a [55], miR-152 [55]) and VEGF upstream transcription factors (STAT3: miR-874 [56], Bmi-1: miR-16 [57], E2F3: miR-34a [58], NF90: miR-590-5p [59]). Conversely, miRNAs targeting the suppressors of these VEGF inducers promoted tumor angiogenesis. Overexpression of miR-21 induced tumor angiogenesis by targeting PTEN, which in turn activated AKT and ERK signaling and finally enhanced HIF-1α and VEGF expression in prostate cancer cells [60]. E-cadherin downregulation by miR-9 activated β-catenin signaling, in turn upregulating VEGF-A expression and promoting tumor angiogenesis [61].

### Tumoral miRNAs regulating VEGF-independent angiogenesis

Several other growth factors also play positive roles in tumor angiogenesis. MiRNAs have been reported to target these pro-angiogenic factors in tumor cells, such as FGF (miR-503 [38]), HDGF (miR-214 [62], miR-497 [63]), angiogenin (miR-409-3p [64]), angiopoietin (miR-204 [65]), and MMPs (miR-9 [66], miR-26b-5p [67], miR-98 [68], miR-181a-5p [69]). Moreover, some miRNAs were shown to simultaneously target more than one angiogenic factors. MiR-190 significantly suppressed tumor angiogenesis via targeting VEGF, HGF and IGF1, thus altering the local microenvironment and subsequently regulating neighboring ECs [31].

Angiogenesis is regulated by the balance between pro- and anti-angiogenic factors. Angiostatin, endostatin and thrombospondin-1 (*TSP-1*) are endogenous anti-angiogenic factors [70–72], among which TSP-1 is most well-known. TSP-1 binds to its receptor CD36 on the surface of ECs, as well as by binding to β1-integrins, leading to cell apoptosis [73]. In addition, TSP-1 inhibits the activity of MMP-9, thereby hindering the release of VEGF-A sequestered in extracellular matrix [74]. Several miRNAs, including miR-17-92 cluster [75], miR-182 [76], miR-194 [77] and miR-467 [78], have been reported to target TSP-1 in tumor cells, thus decreasing TSP-1 secretion and promoting tumor angiogenesis.

## Cell-autonomous regulation of tumor angiogenesis by miRNAs in endothelial cells

The importance of miRNAs in ECs was demonstrated by the silencing of Dicer, an enzyme responsible for miRNA maturation. Knockdown of Dicer in ECs inhibited cell proliferation, migration and cord formation [79], indicating that miRNAs are important in the function of ECs. Many studies have shown that miRNAs in ECs regulate the cellular response to angiogenic factors by targeting surface receptors and signaling molecules (Table 2).

**Table 2:** MiRNAs involved in cell-autonomous regulation of tumor angiogenesis in ECs

	miRNAs	Targets	Function	Ref
pro-angiogenesis	miR-132	p120RasGAP	promote ECs proliferation, tube formation	[86]
	miR-130a	GAX, HOXA5	promote ECs proliferation, migration, tube formation	[87]
	miR-146a	BRCA1	promote ECs proliferation, migration, tube formation	[84]
anti-angiogenesis	miR-128	VEGFR-2	inhibit ECs tube formation	[42]
	miR-497	VEGFR-2	induce ECs apoptosis	[82]
	miR-296	HGS	inhibit ECs tube formation	[83]
	miR-125b	VE-cadherin	inhibit ECs tube formation	[85]
	miR-34a	E2F3, SIRT1, survivin, CDK4	inhibit ECs proliferation, migration and tube formation	[58]
	miR-126	ADM	inhibit ECs migration and tube formation	[88]

*HSG* hepatocyte growth factor-regulated tyrosine kinase substrate, *BRCA1* breast cancer 1, *p120RasGAP* Ras p21 protein activator 1, *SIRT1* sirtuin 1, *ADM* adrenomedullin

### MiRNAs targeting angiogenic factor receptors in endothelial cells

Tyrosine kinase receptors VEGFR and platelet-derived growth factor receptor (PDGFR) are primarily located on the surface of ECs [80, 81]. The binding of VEGF-A to VEGFR-2 and PDGF to PDGFR initiates a cascade of signals that result in endothelial cell proliferation and migration [80]. Several miRNAs have been reported to directly target these receptors in ECs, such as miR-128 [42] and miR-497 [82], blocking their downstream signaling to inhibit tumor angiogenesis. On the contrary, miRNAs targeting the suppressors of these receptors promoted tumor angiogenesis. MiR-296 contributed to angiogenesis by directly targeting the hepatocyte growth factor-regulated tyrosine kinase substrate (HGS) mRNA, thereby reducing HGS-mediated degradation of VEGFR-2 and PDGFRβ in ECs [83]. MiR-146a enhanced tumor angiogenesis by targeting BRCA1 which conferred a transcriptional repression on PDGFRA [84].

### MiRNAs targeting signaling molecules in endothelial cells

MiRNAs have been implicated as key modulators of angiogenic signal transduction pathways. MiR-125b suppressed tube formation of ECs by inhibiting VE-cadherin translation [85]. MiR-34a was shown to inhibit the proliferation, migration and tube formation of ECs by downregulating a number of key proteins including E2F3, SIRT1, survivin and CDK4 [58]. Conversely, miRNAs promote angiogenesis by targeting negative regulators in angiogenic signaling pathways. MiR-132 was highly expressed in the endothelium of human tumors and hemangiomas but was undetectable in normal endothelium. MiR-132 induced Ras activation via suppressing p120RasGAP expression, thus promoting neovascularization [86]. MiR-130a is a positive regulator of ECs largely through targeting anti-angiogenic homeobox gene GAX and HOXA5 [87]. Notably, miR-126 has been well known as an endothelial-specific miRNA and has pro-angiogenic action during embryo development [88]. However, a recent study illustrated an anti-angiogenic role of miR-126 during tumor angiogenesis by targeting a pro-angiogenic gene adrenomedullin (ADM) [89]. This study suggests that the role of miRNAs may vary depending on microenvironment.

## Regulation of tumor angiogenesis by miRNAs in tumor-derived extracellular vesicles

The discovery of extracellular vesicles (EVs) represents an exciting area of research that offers novel mechanisms for intercellular communication. EVs, generally including exosomes (typically 30–100 nm) and microparticles or microvesicles (typically 100 nm-1 μm), are small secretory vesicles that contain proteins, DNA, and coding and non-coding RNAs [90]. EVs can be released from various cell types under physiological and pathological conditions and transfer their cargo to proximal or distal cells [91]. In many published articles, the collective term “microvesicles” was used for all the EVs between 30 and 1000 nm in- diameter.

Recent evidence has demonstrated that cancer cell-derived EVs can be transferred to ECs where they induce pro-angiogenic effects. Glioblastoma cells released exosomes containing mRNA, miRNAs and angiogenic proteins. These exosomes were taken up by brain microvascular endothelial cells and modulated angiogenesis [92]. A set of pro-angiogenic mRNAs and miRNAs were enriched in the microvesicles of CD105-positive renal cancer stem cells, therefore conferring an activated angiogenic phenotype to ECs [93].

MiRNAs are the most abundant non-coding RNAs enriched in EVs, and studies have also showed that EVs contain components of the RNA-induced silencing complex (RISC), suggesting potent regulatory role of EV-miRNA in recipient cells [94]. Several studies have reported the regulation of signaling pathways in ECs by miRNAs delivered via tumor-derived EVs (Table 3). MiR-494 containing EVs secreted by tumor cells were taken up by ECs and promoted the migration of EC via targeting of PTEN and subsequent activation of Akt/eNOS pathway [95]. EV-encapsulated miR-9 transferred from tumor cells to ECs reduced SOCS5 level, leading to JAK-STAT pathway activation to promote endothelial cell migration and sprout [96]. Colorectal cancer cells secreted miR-1246 via EVs, which activated Smad1/5/8 signaling through targeting promyelocytic leukemia (PML) mRNA in recipient ECs, promoting angiogenic activities [97].

**Table 3:** MiRNAs involved in angiogenesis regulation by tumor-derived extracellular vesicles

miRNAs in EVs	Targets	Function	Ref
miR-494	PTEN	promote ECs migration, tube formation	[95]
miR-9	SOCS5	promote ECs migration, tube formation	[96]
miR-1246	PML	promote ECs proliferation, migration, tube formation	[97]
miR-23a	PHD1, PHD2, ZO-1	increase the number of tumor vessels, disrupt endothelial barriers	[98]
miR-210	Ephrin-A3	Promote ECs tube formation	[99]
miR-105	ZO-1	destroy tight junctions and integrity of vascular endothelial barriers	[102]

*SOCS5* suppressor of cytokine signaling 5, *PML* promyelocytic leukemia, *PHD* prolyl hydroxylase, *ZO-1* tight junction protein 1

Hypoxia is a potent trigger of angiogenesis. Some studies have focused on clarifying how hypoxia affects tumor angiogenesis through tumor cell-derived EVs. Hsu et al. found that more exosomes were produced under hypoxic conditions than normoxic conditions by lung cancer cells. Tumor-derived exosomal miR-23a directly suppressed the expression of prolyl- hydroxylase 1 and 2 (PHD1 and 2), leading to the accumulation of HIF-1α in ECs and increased angiogenesis [98]. Tadokoro et al. reported that exosomes derived from leukemia cells under hypoxia are potent inducers of angiogenesis through transfer of miR-210 to ECs, downregulating the expression of receptor tyrosine kinase ligand Ephrin-A3 in HUVECs [99]. Neutral Sphingomyelinase 2 (nSMase2) was shown to regulate exosomal miR-210 secretion by metastatic cancer cells [100].

Tumor-induced vascular vessels commonly have abnormal structure with increased permeability and incomplete cellular junction [101]. Zhou et al. showed that in endothelial monolayers, exosomal miR-105 derived from breast cancer cells efficiently destroyed tight junctions and the integrity of natural vascular endothelial barriers by targeting ZO-1 in ECs, thus promoting breast cancer metastasis [102]. Similarly, lung cancer cells-secreted exosomal miR-23a also inhibited tight junction protein ZO-1, thereby increasing vascular permeability and cancer trans-endothelial migration [98]. Collectively, these observations suggest that tumor-secreted EV miRNAs participate in intercellular communication and function as a novel pro-angiogenic mechanism.

## MiRNAs as biomarkers for anti-angiogenic therapy response

As angiogenesis is essential for tumor growth and progression, anti-angiogenic therapies have been developed to improve the outcomes of cancer patients. Several agents, including bevacizumab, aflibercept and most recently ramucirumab that target the VEGF/VEGFR signaling pathway, have been approved across some cancer types. In addition, multi-targeted tyrosine kinase inhibitors (TKIs), such as sorafenib and sunitinib, also block angiogenesis signaling. However, the overall response rate of these anti-angiogenic therapies is unsatisfactory in the clinic. Therefore, selecting out the patients, who would benefit from anti-angiogenic treatment, will help to promote therapy efficacy and avoid unnecessary toxic adverse effect. Due to the high tissue specificity and stability of miRNAs and their altered expression in tumor angiogenesis, miRNAs have been suggested as potent predictive biomarkers for therapeutic response (Table 4).

**Table 4:** Tissue and blood miRNA biomarkers for anti-angiogenesis therapeutic response

Clinical Outcomes	miRNAs	Sample type	Detection time	Therapy	Cancer	Patient cohort^a^	Ref
Better outcomes	miR-378	tissue	post-therapy	bevacizumab	ovarian cancer	113/34	[104]
	miR-455-5p	tissue	post-therapy	bevacizumab	mCRC	212/121	[105]
	miR-221	tissue	post-therapy	sunitinib	mRCC	30/27	[107]
	miR-155, miR-484	tissue	post-therapy	sunitinib	mRCC	16/63	[108]
	miR-126	blood	pre- and post-therapy	bevacizumab	mCRC	75/68	[111]
	miR-665	blood	pre-therapy	bevacizumab/erlotinib	NSCLC	51/50	[112]
Worse outcomes	miR-664-3p,	tissue	post-therapy	bevacizumab	mCRC	212/121	[105]
	miR-425-3p	tissue	pre- and post-therapy	sorafenib	HCC	26/58	[106]
	miR-223	blood	pre-therapy	bevacizumab/erlotinib	NSCLC	51/50	[112]

*mCRC* metastatic colorectal cancer, *HCC* hepatocellular carcinoma, *mRCC* metastatic renal cell carcinoma, *NSCLC* non-small-cell lung cancer

^a^The pattern A/B represents patient number in training cohort and validation cohort respectively

### MiRNA biomarkers of anti-angiogenic therapy response in tumor tissue

Bevacizumab, a humanized monoclonal antibody against VEGF-A, has been approved as combination therapy or monotherapy for first- and second-line treatment of several advanced cancers, including metastatic colorectal cancer (mCRC), metastatic renal cell carcinoma (mRCC), metastatic non-small cell lung cancer (NSCLC), progressive glioblastoma, metastatic cervical cancer and ovarian cancer etc. [103]. In TCGA dataset, high miR-378 expression was associated with poor progression-free survival (PFS) in recurrent ovarian cancer patients treated with bevacizumab (low vs. high, 9.2 vs. 4.2 months; *p* = 0.04). Multivariate analysis revealed that miR-378 expression was an independent predictor for PFS after anti-angiogenic treatment [104]. For patients with mCRC treated with first line CAPEOX (capecitabine and oxaliplatin) alone, or CAPEOX and bevacizumab (CAPEOXBEV), predictive miRNAs for therapy effectiveness were identified using PCR arrays. Higher miR-664-3p level and lower miR-455-5p level were predictive of improved outcome in the CAPEOXBEV cohorts and showed a significant interaction with bevacizumab effectiveness [105].

Several multi-target TKIs have potent anti-angiogenic effects due to the inhibition of VEGFR and PDGFR, and have been approved, including sorafenib for mRCC and unresectable hepatocellular carcinoma (HCC), and sunitinib for mRCC. High level of miR-425-3p was associated with longer PFS in HCC patients treated with sorafenib. Multivariate analysis confirmed the predictive significance of miR-425-3p, suggesting that assessment of miR-425-3p levels in liver biopsies could help in stratifying patients with advanced HCC for sorafenib treatment [106]. Khella et al. compared miRNA profile between patients with a short (≤12 months) versus prolonged (> 12 months) PFS under sunitinib as first-line therapy for metastatic RCC. They developed miRNA statistical models that could accurately distinguish the two groups. MiR-221 overexpression was validated to be associated with a poor PFS, while its target VEGFR2 was associated with longer survival [107]. In another study, miRNA profiling in tumor tissues of sunitinib-treated mRCC patients was performed by TaqMan Low Density Arrays. Decreased tissue levels of miR-155 and miR-484 were significantly associated with increased time to progression (TTP), suggesting miR-155 and miR-484 are potentially connected with sunitinib resistance and failure of the therapy [108].

### Circulating miRNA biomarkers of anti-angiogenic therapy response

Although tissue miRNA expression could serve as molecular classifier for clinical outcome, there are several drawbacks including small quantities of diagnostic tumor tissue, genetic tumor heterogeneity in space and time, and unavailable acquisition of repeated biopsies. Therefore, circulating biomarkers offer a non-invasive opportunity for early diagnosis, prognosis evaluation and drug response prediction in cancer. Circulating miRNAs are stable and reproducible in blood due to their incorporation in exosomes/microvesicles or protein complexes, protecting them from degradation by endogenous RNase [109, 110]. Technical advances in detection and profiling makes them promising candidates. Recently, the occurrence of miRNAs in the serum or plasma of human has been repeatedly observed and become the focus of biomarker discovery.

Hansen et al. investigated the predictive value of circulating miR-126 in mCRC patients receiving first-line chemotherapy combined with bevacizumab. Blood samples of patients were collected before treatment (baseline), 3 weeks after treatment and at progression. Evaluation of miR-126 expression in plasma showed that non-responders had an increase in miR-126 level relative to baseline, while responders with decreased miR-126 alteration. The results indicated that changes in circulating miR-126 during treatment represented a possible biomarker for the response to anti-angiogenic containing therapy [111]. M. Joerger’s study assessed the predictive value of circulating miRNA in patients with non-squamous NSCLC, receiving treatment with first-line bevacizumab and erlotinib followed by platinum-based chemotherapy at progression. MiRNA profiling in pre-treatment blood showed that 12 miRNAs were significantly associated with tumor shrinkage following bevacizumab/erlotinib treatment, with miR-665 being the strongest predictive marker. Patients with high miR-665 expression had a higher chance for tumor shrinkage. Furthermore, miR-223 was found to be associated with both TTP following bevacizumab/erlotinib treatment and TTP following second-line chemotherapy [112]. Collectively, these studies indicate that tissue and circulating miRNAs could serve as predictive biomarkers for response to anti-angiogenic therapy.

## MiRNA-based therapeutics to target tumor angiogenesis

Given the important regulatory role of miRNAs in tumor angiogenesis, targeting or delivering miRNAs to tumors holds great promise as a novel therapeutic approach. In this case, it is essential to deliver antagomiRs or miRNA mimics into tumor cells or endothelial cells. As naked miRNAs are hydrophilicity, unable to pass through cell membranes, and could be easily degraded by serum RNase [113], in vivo application of miRNA requires formulation into delivery systems. Here we summarize some miRNA delivery approaches that have been applied to suppress angiogenesis in vivo (Table 5).

**Table 5:** MiRNA-based therapeutics to target tumor angiogenesis

miRNAs	Delivery systems	Cancer	Targets	Ref
miR-125b	PEI	lung cancer, colorectal cancer	VE-cadherin	[85]
miR-192	DOPC	ovarian cancer, renal tumor	EGR1, HOXB9	[116]
miR-200	DOPC	lung cancer, ovarian cancer, renal cancer	IL8, CXCL1	[117]
miR-520d	DOPC	ovarian cancer	EphA2	[118]
miR-132	cRGD	breast cancer	p120RasGAP	[86]
miR-499	APRPG-PEG	colorectal cancer	VEGF	[122]
miR-29a/c	CMVs	gastric cancer	VEGF	[127]
miR-150	CMVs	sarcoma	VEGF	[128]

*PEI* polymer polyethylenimine, *DOPC* 1,2-dioleoyl-sn-glycero-3-phosphatidylcholine, *cRGD* cyclicArginine-Glycine-Aspartic acid, *APRPG-PEG* Ala-Pro-Arg-Pro-Glypeptide-conjugated polyethyleneglycol, *CMVs* Cell-derived membrane vesicles

### Nanoparticles for therapeutic miRNA delivery

Nanoparticle delivery systems, including viral and non-viral ones, have been addressed to protect miRNAs from degradation by serum nucleases when administered systemically [114]. The advantage of non-viral approach is safety and avoiding induction of toxic immune response compared with viral delivery. Non-viral delivery systems mainly use liposomes, lipoplexes and polyplexes as miRNA carriers. The encapsulation of miRNAs into polycation liposomes has been well studied. Muramatsu et al. injected miR-125b directly into the subcutaneous tumor using non-viral vectors composed of the cationic polymer polyethylenimine (PEI). Administration of miR-125b induced formation of non-functional blood vessels and inhibited in vivo tumor growth by targeting VE-cadherin, suggestive of therapeutic potential [85]. Another kind of liposome is neutral liposomalparticle 1,2-dioleoyl- sn-glycero-3-phosphatidylcholine (DOPC) [115]. Wu et al. reported that the delivery of miR-192, which targets EGR1 and HOXB9, to tumors using the DOPC nanoliposomes significantly inhibited tumor angiogenesis of multiple ovarian and renal cancer models [116]. This delivery platform has also been used for miR-200 and miR-520d in several cancer types in preclinical models to inhibit tumor angiogenesis in vivo [117, 118].

To turn miRNA into therapeutics, it is critical to deliver antagomiRs or miRNA mimics specifically into target cells. Endothelial cells in the tumor microenvironment have been actively targeted in vivo for miRNA delivery. The cyclic Arginine-Glycine-Aspartic acid (cRGD) peptide coupled nanoparticle is most widely used. The cRGD could bind to integrin αvβ3 and αvβ5, which are present on tumor ECs, as well as on certain tumor cells. Studies have reported that systemic administration of anti-miR-296, anti-miRNA-132 or miR-7 mimics using the cRGD modified nanoparticles strongly reduced angiogenesis and tumor burden in mice, offering promise for miRNA-based anti-tumor therapeutic [86, 119, 120]. In addition to cRGD modification, anti-angiogenic effect was achieved by using miRNA- incoporated nanoparticles bearing Ala-Pro-Arg-Pro-Gly (APRPG) peptide as well. APRPG has an affinity for VEGFR-1, which is overexpressed on ECs and certain cancer cells [121]. In vivo delivery of miR-499 via APRPG-PEG-modified lipoplexes accumulated in both angiogenic vessels and cancer cells, resulting in the downregulation of miR-499 targeted proteins and inhibition of tumor growth and angiogenesis [122].

### Cell-derived membrane vesicles for therapeutic miRNA delivery

Cell-derived membrane vesicles (CMVs), including exosomes and microvesicles, are endogenous carriers transporting biological molecules to recipient cells. In contrast to established synthetic nanoparticles, CMVs are natural transporters and hence, less likely to exert toxicity or immune response, which probably due to their lowhoming to the liver [123, 124]. Moreover, CMVs are shown to contain ribonucleoprotein involved in cellular RNA transport as well as RISC components. Exosomes have already been utilized to transfer therapeutic RNAi to treat diseases [125, 126]. Therefore, CMVs hold great promise as a novel class of miRNA delivery system.

Zhang et al. revealed that CMVs derived from HEK293T cells are capable of packing and transporting miR-29a/c into gastric cancer cells. The in vivo experiments illustrated that CMVs could stably transfer miR-29a/c into the implanted tumor cells in mice, playing anti- angiogenic role by directly targeting VEGF [127]. Their data proved that CMVs function as a potential carrier of miRNAs for targeted therapy in gastric cancer. Similarly, Liu et al. used a MV-delivered antisense RNA against miRNA to treat tumors. With the utilization of CMVs derived from HEK293T cells, they transferred anti-miR-150 into mice and found that the neutralization of miR-150 downregulated VEGF levels in vivo and attenuates angiogenesis and tumor growth [128]. These results propose a novel anti-cancer strategy using miRNA- containing CMVs to control tumor angiogenesis.

## Conclusions

Significant advances have been made in exploring the regulatory role of miRNAs in tumor angiogenesis. The rapidly increasing discoveries shall pave the way in the use of miRNAs as predictive biomarkers for anti-angiogenic treatments and as miRNA-based strategy against tumor angiogenesis in the future, though there are some challenges.

Given the low response rate of anti-angiogenic agents, a step forward would be discovering predictive biomarkers to distinguish responders from non-responders. So far, several candidate miRNA biomarkers have been identified, but they emerged from small studies and should be confirmed in large prospective trials. Besides, there has been no unified control miRNAs for normalization in the analysis of circulating miRNA expression. A proposed solution to this problem may be the introduction of exogenous control [129].

Multi-targeted anti-angiogenic approach might exert increasing antitumor efficacy. Since a single miRNA has the potential to regulate angiogenesis by targeting multiple mRNAs, miRNA holds great promise as therapeutic approach for tumor angiogenesis treatment [130]. However, targeting miRNAs systemically may affect normal angiogenesis in which these miRNAs might play regulatory roles as well. In this regard, it is important to identify and target miRNAs that could distinguish angiogenic endothelial cells in tumor vasculature from those in normal tissues [131, 132], thus achieving more specific therapeutic effect and reducing side effect.

At the same time, developing technologies to deliver miRNAs to specific cells in vivo is highly essential for their therapeutic application. Despite having many strategies reported, the successful delivery approach in vivo is still limited. Clinical translation has been hampered by dose-dependent toxicity upon systemic administration [133, 134]. Intriguingly, Kalluri’s recent study showed that exosomes derived from normal fibroblast-like mesenchymal cells exhibited a superior ability to deliver RNAi to suppress pancreatic cancer growth, and showed minimum cytotoxic effects in vivo compared to synthetic nanoparticles. The engineered exosomes (known as iExosomes) target oncogenic KRAS with enhanced efficacy probably due to that CD47 on exosomes surface contributed to the evasion from immune clearance in the circulation, and KRAS-stimulated macropinocytosis increased pancreatic cancer cells uptake of iExosomes [126]. This study offers insight into the therapeutic potential of exosomes in the delivery of nucleic acid-based drugs. However, in terms of using exosomes for specific targeting of angiogenesis-related miRNAs in tumor cells or ECs, there are several aspects to consider. New targeting moieties should be tested for selective homing of exosomes to certain tumor cells and tumor-associated ECs. New targeting moieties, such as peptides and antibody fragments, could be cloned into exosomal surface proteins, or directly attached to the surface of exosomes through chemical conjugation, therefore bypassing time-consuming cloning procedures. Furthermore, the use of miRNA sponge would allow for persistent suppression of miRNA in target tissues following exosome delivery. Additionally, it is critical to find a robust cell source that produces high quantities of exosomes for use in miRNA delivery. For instance, Myc-immortalized human ES-derived mesenchymal stem cells (MSCs) have been demonstrated as a scalable source for production of therapeutic exosomes for regenerative medicine [135].

In summary, better understanding of the regulatory network of miRNAs in tumor angiogenesis provides new insights in the biological process of tumor-associated neovasculature. Advances in profiling and delivery system offer clinical translation potential of miRNAs as predictive biomarkers for anti-angiogenic therapy response and therapeutics against tumor angiogenesis.

## References

[1] Hanahan D, Weinberg RA (2011). Hallmarks of cancer: the next generation. Cell.

[2] Lee SL (2009). Hypoxia-induced pathological angiogenesis mediates tumor cell dissemination, invasion, and metastasis in a zebrafish tumor model. Proc Natl Acad Sci U S A.

[3] Wang Z (2015). Broad targeting of angiogenesis for cancer prevention and therapy. Semin Cancer Biol.

[4] Lathia JD (2011). Deadly teamwork: neural cancer stem cells and the tumor microenvironment. Cell Stem Cell.

[5] Calabrese C (2007). A perivascular niche for brain tumor stem cells. Cancer Cell.

[6] Herbert SP, Stainier DY (2011). Molecular control of endothelial cell behaviour during blood vessel morphogenesis. Nat Rev Mol Cell Biol.

[7] Hanahan D, Folkman J (1996). Patterns and emerging mechanisms of the angiogenic switch during tumorigenesis. Cell.

[8] Baeriswyl V, Christofori G (2009). The angiogenic switch in carcinogenesis. Semin Cancer Biol.

[9] Bergers G, Benjamin LE (2003). Tumorigenesis and the angiogenic switch. Nat Rev Cancer.

[10] Bartel DP (2009). MicroRNAs: target recognition and regulatory functions. Cell.

[11] Wang W, Zhang E, Lin C (2015). MicroRNAs in tumor angiogenesis. Life Sci.

[12] Calin GA, Croce CM (2006). MicroRNA signatures in human cancers. Nat Rev Cancer.

[13] Martello G (2010). A MicroRNA targeting dicer for metastasis control. Cell.

[14] Xie T (2016). MicroRNAs as regulators, biomarkers and therapeutic targets in the drug resistance of colorectal cancer. Cell Physiol Biochem.

[15] Thery C (2011). Exosomes: secreted vesicles and intercellular communications. F1000 Biol Rep.

[16] Ferrara N, Gerber HP, LeCouter J (2003). The biology of VEGF and its receptors. Nat Med.

[17] Roskoski R (2007). Vascular endothelial growth factor (VEGF) signaling in tumor progression. Crit Rev Oncol Hematol.

[18] Ferrara N (2009). VEGF-A: a critical regulator of blood vessel growth. Eur Cytokine Netw.

[19] Claesson-Welsh L (2008). VEGF-B taken to our hearts: specific effect of VEGF-B in myocardial ischemia. Arterioscler Thromb Vasc Biol.

[20] Linderholm B (1998). Vascular endothelial growth factor is of high prognostic value in node-negative breast carcinoma. J Clin Oncol.

[21] Lei Z (2009). Regulation of HIF-1alpha and VEGF by miR-20b tunes tumor cells to adapt to the alteration of oxygen concentration. PLoS One.

[22] Chou J (2013). GATA3 suppresses metastasis and modulates the tumour microenvironment by regulating microRNA-29b expression. Nat Cell Biol.

[23] Long J (2010). Identification of microRNA-93 as a novel regulator of vascular endothelial growth factor in hyperglycemic conditions. J Biol Chem.

[24] Li F (2014). Role of microRNA-93 in regulation of angiogenesis. Tumour Biol.

[25] Yang IP (2012). MicroRNA-93 inhibits tumor growth and early relapse of human colorectal cancer by affecting genes involved in the cell cycle. Carcinogenesis.

[26] Zhu X (2012). miR-126 enhances the sensitivity of non-small cell lung cancer cells to anticancer agents by targeting vascular endothelial growth factor a. Acta Biochim Biophys Sin Shanghai.

[27] Liu B (2009). MiR-126 restoration down-regulate VEGF and inhibit the growth of lung cancer cell lines in vitro and in vivo. Lung Cancer.

[28] Zhu N (2011). Endothelial-specific intron-derived miR-126 is down-regulated in human breast cancer and targets both VEGFA and PIK3R2. Mol Cell Biochem.

[29] Sasahira T (2012). Downregulation of miR-126 induces angiogenesis and lymphangiogenesis by activation of VEGF-A in oral cancer. Br J Cancer.

[30] Chen H (2014). Reduced miR-126 expression facilitates angiogenesis of gastric cancer through its regulation on VEGF-A. Oncotarget.

[31] Hao Y (2014). The synergistic regulation of VEGF-mediated angiogenesis through miR-190 and target genes. RNA.

[32] Wang R (2013). MicroRNA-195 suppresses angiogenesis and metastasis of hepatocellular carcinoma by inhibiting the expression of VEGF, VAV2, and CDC42. Hepatology.

[33] Choi YC (2011). Regulation of vascular endothelial growth factor signaling by miR-200b. Mol Cells.

[34] Zhang HF, Xu LY, Li EM (2014). A family of pleiotropically acting microRNAs in cancer progression, miR-200: potential cancer therapeutic targets. Curr Pharm Des.

[35] Zhu X (2013). miR-203 suppresses tumor growth and angiogenesis by targeting VEGFA in cervical cancer. Cell Physiol Biochem.

[36] Wang W (2014). MicroRNA-497 suppresses angiogenesis by targeting vascular endothelial growth factor a through the PI3K/AKT and MAPK/ERK pathways in ovarian cancer. Oncol Rep.

[37] Yan JJ (2015). MiR-497 suppresses angiogenesis and metastasis of hepatocellular carcinoma by inhibiting VEGFA and AEG-1. Oncotarget.

[38] Zhou B (2013). MicroRNA-503 targets FGF2 and VEGFA and inhibits tumor angiogenesis and growth. Cancer Lett.

[39] Cheng J (2016). Downregulation of miRNA-638 promotes angiogenesis and growth of hepatocellular carcinoma by targeting VEGF. Oncotarget.

[40] Kumar B (2011). VEGF-C differentially regulates VEGF-A expression in ocular and cancer cells; promotes angiogenesis via RhoA mediated pathway. Angiogenesis.

[41] Ye J (2013). miRNA-27b targets vascular endothelial growth factor C to inhibit tumor progression and angiogenesis in colorectal cancer. PLoS One.

[42] Hu J (2014). microRNA-128 plays a critical role in human non-small cell lung cancer tumourigenesis, angiogenesis and lymphangiogenesis by directly targeting vascular endothelial growth factor-C. Eur J Cancer.

[43] Yamakuchi M (2011). MicroRNA-22 regulates hypoxia signaling in colon cancer cells. PLoS One.

[44] Yamakuchi M (2010). P53-induced microRNA-107 inhibits HIF-1 and tumor angiogenesis. Proc Natl Acad Sci U S A.

[45] Cha ST (2010). MicroRNA-519c suppresses hypoxia-inducible factor-1alpha expression and tumor angiogenesis. Cancer Res.

[46] Zhang H (2014). MicroRNA-145 inhibits the growth, invasion, metastasis and angiogenesis of neuroblastoma cells through targeting hypoxia-inducible factor 2 alpha. Oncogene.

[47] Chai ZT (2013). MicroRNA-26a inhibits angiogenesis by down-regulating VEGFA through the PIK3C2alpha/Akt/HIF-1alpha pathway in hepatocellular carcinoma. PLoS One.

[48] Yin Y (2013). Downregulation of miR-145 associated with cancer progression and VEGF transcriptional activation by targeting N-RAS and IRS1. Biochim Biophys Acta.

[49] Zheng Y (2013). The role of miR-18a in gastric cancer angiogenesis. Epatogastroenterology.

[50] Shi ZM (2012). MiR-128 inhibits tumor growth and angiogenesis by targeting p70S6K1. PLoS One.

[51] Xu Q (2012). MiR-145 directly targets p70S6K1 in cancer cells to inhibit tumor growth and angiogenesis. Nucleic Acids Res.

[52] Guan B (2017). Tumor-suppressive microRNA-218 inhibits tumor angiogenesis via targeting the mTOR component RICTOR in prostate cancer. Oncotarget.

[53] Png KJ (2011). A microRNA regulon that mediates endothelial recruitment and metastasis by cancer cells. Nature.

[54] Shi ZM (2013). MiRNA-181b suppresses IGF-1R and functions as a tumor suppressor gene in gliomas. RNA.

[55] Xu Q (2013). A regulatory circuit of miR-148a/152 and DNMT1 in modulating cell transformation and tumor angiogenesis through IGF-IR and IRS1. J Mol Cell Biol.

[56] Zhang X (2015). miR-874 functions as a tumor suppressor by inhibiting angiogenesis through STAT3/VEGF-A pathway in gastric cancer. Oncotarget.

[57] Chen F (2016). Up-regulation of microRNA-16 in Glioblastoma inhibits the function of endothelial cells and tumor angiogenesis by targeting Bmi-1. Anti Cancer Agents Med Chem.

[58] Kumar B (2012). Dysregulation of microRNA-34a expression in head and neck squamous cell carcinoma promotes tumor growth and tumor angiogenesis. PLoS One.

[59] Zhou Q (2016). MiR-590-5p inhibits colorectal cancer angiogenesis and metastasis by regulating nuclear factor 90/vascular endothelial growth factor a axis. Cell Death Dis.

[60] Liu LZ (2011). MiR-21 induced angiogenesis through AKT and ERK activation and HIF-1alpha expression. PLoS One.

[61] Ma L (2010). miR-9, a MYC/MYCN-activated microRNA, regulates E-cadherin and cancer metastasis. Nat Cell Biol.

[62] Shih TC (2012). MicroRNA-214 downregulation contributes to tumor angiogenesis by inducing secretion of the hepatoma-derived growth factor in human hepatoma. J Hepatol.

[63] Zhao WY (2013). Downregulation of miR-497 promotes tumor growth and angiogenesis by targeting HDGF in non-small cell lung cancer. Biochem Biophys Res Commun.

[64] Weng C (2012). miR-409-3p inhibits HT1080 cell proliferation, vascularization and metastasis by targeting angiogenin. Cancer Lett.

[65] Flores-Perez A (2016). Dual targeting of ANGPT1 and TGFBR2 genes by miR-204 controls angiogenesis in breast cancer. Sci Rep.

[66] Zhang H (2012). microRNA-9 targets matrix metalloproteinase 14 to inhibit invasion, metastasis, and angiogenesis of neuroblastoma cells. Mol Cancer Ther.

[67] Wang Y (2016). Regulation of proliferation, angiogenesis and apoptosis in hepatocellular carcinoma by miR-26b-5p. Tumour Biol.

[68] Siragam V (2013). MicroRNA miR-98 inhibits tumor angiogenesis and invasion by targeting activin receptor-like kinase-4 and matrix metalloproteinase-11. Oncotarget.

[69] Li Y (2015). miR-181a-5p inhibits cancer cell migration and angiogenesis via Downregulation of matrix Metalloproteinase-14. Cancer Res.

[70] O'Reilly MS (1994). Angiostatin: a novel angiogenesis inhibitor that mediates the suppression of metastases by a Lewis lung carcinoma. Cell.

[71] O'Reilly MS (1997). Endostatin: an endogenous inhibitor of angiogenesis and tumor growth. Cell.

[72] Dameron K (1994). Control of angiogenesis in fibroblasts by p53 regulation of thrombospondin-1. Science.

[73] Dawson DW (1997). CD36 mediates the in vitro inhibitory effects of thrombospondin-1 on endothelial cells. J Cell Biol.

[74] Rodriguez-Manzaneque JC (2001). Thrombospondin-1 suppresses spontaneous tumor growth and inhibits activation of matrix metalloproteinase-9 and mobilization of vascular endothelial growth factor. Proc Natl Acad Sci U S A.

[75] Dews M (2006). Augmentation of tumor angiogenesis by a Myc-activated microRNA cluster. Nat Genet.

[76] Amodeo V (2013). Effects of anti-miR-182 on TSP-1 expression in human colon cancer cells: there is a sense in antisense?. Expert Opin Ther Targets.

[77] Sundaram P (2011). p53-responsive miR-194 inhibits thrombospondin-1 and promotes angiogenesis in colon cancers. Cancer Res.

[78] Bhattacharyya S (2012). Novel tissue-specific mechanism of regulation of angiogenesis and cancer growth in response to hyperglycemia. J Am Heart Assoc.

[79] Suarez Y (2007). Dicer dependent microRNAs regulate gene expression and functions in human endothelial cells. Circ Res.

[80] Kerbel RS (2008). Tumor angiogenesis. N Engl J Med.

[81] Zhang J (2009). Differential roles of PDGFR-alpha and PDGFR-beta in angiogenesis and vessel stability. FASEB J.

[82] Tu Y (2015). Overexpression of miRNA-497 inhibits tumor angiogenesis by targeting VEGFR2. Sci Rep.

[83] Wurdinger T (2008). miR-296 regulates growth factor receptor overexpression in angiogenic endothelial cells. Cancer Cell.

[84] Zhu K (2013). MiR-146a enhances angiogenic activity of endothelial cells in hepatocellular carcinoma by promoting PDGFRA expression. Carcinogenesis.

[85] Muramatsu F (2013). microRNA-125b inhibits tube formation of blood vessels through translational suppression of VE-cadherin. Oncogene.

[86] Anand S (2010). MicroRNA-132-mediated loss of p120RasGAP activates the endothelium to facilitate pathological angiogenesis. Nat Med.

[87] Chen Y, Gorski DH (2008). Regulation of angiogenesis through a microRNA (miR-130a) that down-regulates antiangiogenic homeobox genes GAX and HOXA5. Blood.

[88] Fish JE (2008). miR-126 regulates angiogenic signaling and vascular integrity. Dev Cell.

[89] Huang TH, Chu TY (2014). Repression of miR-126 and upregulation of adrenomedullin in the stromal endothelium by cancer-stromal cross talks confers angiogenesis of cervical cancer. Oncogene.

[90] Raposo G, Stoorvogel W (2013). Extracellular vesicles: exosomes, microvesicles, and friends. J Cell Biol.

[91] Desrochers LM, Antonyak MA, Cerione RA (2016). Extracellular vesicles: satellites of information transfer in cancer and stem cell biology. Dev Cell.

[92] Skog J (2008). Glioblastoma microvesicles transport RNA and proteins that promote tumour growth and provide diagnostic biomarkers. Nat Cell Biol.

[93] Grange C (2011). Microvesicles released from human renal cancer stem cells stimulate angiogenesis and formation of lung premetastatic niche. Cancer Res.

[94] Gibbings D (2009). Multivesicular bodies associate with components of miRNA effector complexes and modulate miRNA activity. Nat Cell Biol.

[95] Mao G (2015). Tumor-derived microRNA-494 promotes angiogenesis in non-small cell lung cancer. Angiogenesis.

[96] Zhuang G (2012). Tumour-secreted miR-9 promotes endothelial cell migration and angiogenesis by activating the JAK-STAT pathway. EMBO J.

[97] Yamada N (2014). Colorectal cancer cell-derived microvesicles containing microRNA-1246 promote angiogenesis by activating Smad 1/5/8 signaling elicited by PML down-regulation in endothelial cells. Biochim Biophys Acta.

[98] Hsu YL, et al. Hypoxic lung cancer-secreted exosomal miR-23a increased angiogenesis and vascular permeability by targeting prolyl hydroxylase and tight junction protein ZO-1. Oncogene. 2017;36(34):4929–942. 10.1038/onc.2017.105 28436951

[99] Tadokoro H (2013). Exosomes derived from hypoxic leukemia cells enhance tube formation in endothelial cells. J Biol Chem.

[100] Kosaka N (2013). Neutral sphingomyelinase 2 (nSMase2)-dependent exosomal transfer of angiogenic microRNAs regulate cancer cell metastasis. J Biol Chem.

[101] Jain RK (2005). Normalization of tumor vasculature: an emerging concept in antiangiogenic therapy. Science.

[102] Zhou W (2014). Cancer-secreted miR-105 destroys vascular endothelial barriers to promote metastasis. Cancer Cell.

[103] Zhao Y, Adjei AA (2015). Targeting angiogenesis in cancer therapy: moving beyond vascular endothelial growth factor. Oncologist.

[104] Chan JK (2014). MiR-378 as a biomarker for response to anti-angiogenic treatment in ovarian cancer. Gynecol Oncol.

[105] Boisen MK (2014). Tissue microRNAs as predictors of outcome in patients with metastatic colorectal cancer treated with first line Capecitabine and Oxaliplatin with or without Bevacizumab. PLoS One.

[106] Vaira V (2015). MicroRNA-425-3p predicts response to sorafenib therapy in patients with hepatocellular carcinoma. Liver Int.

[107] Khella HW (2015). miR-221/222 are involved in response to Sunitinib treatment in metastatic renal cell carcinoma. Mol Ther.

[108] Merhautova J (2015). miR-155 and miR-484 are associated with time to progression in metastatic renal cell carcinoma treated with Sunitinib. Biomed Res Int.

[109] Mitchell PS (2008). Circulating microRNAs as stable blood-based markers for cancer detection. Proc Natl Acad Sci U S A.

[110] Kosaka N, Iguchi H, Ochiya T (2010). Circulating microRNA in body fluid: a new potential biomarker for cancer diagnosis and prognosis. Cancer Sci.

[111] Hansen TF (2015). Changes in circulating microRNA-126 during treatment with chemotherapy and bevacizumab predicts treatment response in patients with metastatic colorectal cancer. Br J Cancer.

[112] Joerger M (2014). Circulating microRNA profiling in patients with advanced non-squamous NSCLC receiving bevacizumab/erlotinib followed by platinum-based chemotherapy at progression (SAKK 19/05). Lung Cancer.

[113] Wang J (2010). Delivery of siRNA therapeutics: barriers and carriers. AAPS J.

[114] Garzon R, Marcucci G, Croce CM (2010). Targeting microRNAs in cancer: rationale, strategies and challenges. Nat Rev Drug Discov.

[115] Landen C (2005). Therapeutic EphA2 gene targeting in vivo using neutral liposomal small interfering RNA delivery. Cancer Res.

[116] Wu SY (2016). A miR-192-EGR1-HOXB9 regulatory network controls the angiogenic switch in cancer. Nat Commun.

[117] Pecot CV (2013). Tumour angiogenesis regulation by the miR-200 family. Nat Commun.

[118] Nishimura M (2013). Therapeutic synergy between microRNA and siRNA in ovarian cancer treatment. Cancer Discov.

[119] Liu XQ (2011). Targeted delivery of antisense inhibitor of miRNA for antiangiogenesis therapy using cRGD-functionalized nanoparticles. Mol Pharm.

[120] Babae N (2014). Systemic miRNA-7 delivery inhibits tumor angiogenesis and growth in murine xenograft glioblastoma. Oncotarget.

[121] Talagas M (2013). VEGFR1 and NRP1 endothelial expressions predict distant relapse after radical prostatectomy in clinically localized prostate cancer. Anticancer Res.

[122] Ando H (2014). Advanced cancer therapy by integrative antitumor actions via systemic administration of miR-499. J Control Release.

[123] El Andaloussi S (2013). Exosomes for targeted siRNA delivery across biological barriers. Adv Drug Deliv Rev.

[124] Zhuang X (2011). Treatment of brain inflammatory diseases by delivering exosome encapsulated anti-inflammatory drugs from the nasal region to the brain. Mol Ther.

[125] Alvarez-Erviti L (2011). Delivery of siRNA to the mouse brain by systemic injection of targeted exosomes. Nat Biotechnol.

[126] Kamerkar S (2017). Exosomes facilitate therapeutic targeting of oncogenic KRAS in pancreatic cancer. Nature.

[127] Zhang H (2016). Cell-derived microvesicles mediate the delivery of miR-29a/c to suppress angiogenesis in gastric carcinoma. Cancer Lett.

[128] Liu Y (2013). Microvesicle-delivery miR-150 promotes tumorigenesis by up-regulating VEGF, and the neutralization of miR-150 attenuate tumor development. Protein Cell.

[129] Gilsbach R (2006). Comparison of in vitro and in vivo reference genes for internal standardization of real-time PCR data. BioTechniques.

[130] Maroof H (2014). Role of microRNA-34 family in cancer with particular reference to cancer angiogenesis. Exp Mol Pathol.

[131] Caporali A, Emanueli C (2011). MicroRNA regulation in angiogenesis. Vasc Pharmacol.

[132] Hong L (2013). Angiogenesis-related microRNAs in colon cancer. Expert Opin Biol Ther.

[133] Kuninty PR (2016). MicroRNA targeting to modulate tumor microenvironment. Front Oncol.

[134] Zhang Y, Satterlee A, Huang L (2012). In vivo gene delivery by nonviral vectors: overcoming hurdles?. Mol Ther.

[135] Chen TS (2011). Enabling a robust scalable manufacturing process for therapeutic exosomes through oncogenic immortalization of human ESC-derived MSCs. J Transl Med.

